# Nano copper oxide catalyzed synthesis of symmetrical diaryl sulfides under ligand free conditions

**DOI:** 10.3762/bjoc.7.101

**Published:** 2011-06-30

**Authors:** K Harsha Vardhan Reddy, V Prakash Reddy, A Ashwan Kumar, G Kranthi, YVD Nageswar

**Affiliations:** 1 Organic Chemistry Divison-I, Indian Institute of Chemical Technology, Hyderabad-500 607, India

**Keywords:** aryl halides, aryl sulfides, copper oxide, cross-coupling, ligand free, potassium thiocyanate, recyclable

## Abstract

Potassium thiocyanate acts as an efficient sulfur surrogate in C–S cross-coupling reactions mediated by recyclable copper oxide nanoparticles under ligand free conditions. This protocol avoids foul smelling thiols, for the synthesis of a variety of symmetrical diaryl sulfides, via the cross-coupling of different aryl halides with potassium thiocyanate, affording corresponding products in moderate to excellent yields.

## Introduction

After the discovery of copper-promoted Ullmann reaction [1–3] for the construction of carbon-hetero atom bonds, several protocols have been reported over the years to establish C–N, C–O, and C–S linkages. Carbon–sulfur bonds are widespread, occurring in numerous pharmaceutically and biologically active compounds [4–8]. A large variety of aryl sulfides are in use for diverse clinical applications in the treatment of cancer [9], HIV [10–11], Alzheimer’s and Parkinson’s diseases [12].

During the last decades, transition and boron group metals, such as palladium [13–18], nickel [19–20], copper [21–37], iron [38], indium [39–40], lanthanum [41] and cobalt [42] have been used in cross-coupling reactions, developed for the formation of carbon–sulfur bonds. Most of these coupling protocols involve the reaction between thiols and aryl halides, resulting in the formation of C–S bonds. Most of these metal-catalyzed reactions involve volatile and foul-smelling thiols as the main drawback, which leads to environmental and safety problems. To overcome these problems, Zhou [43] and coworkers recently reported an efficient C–S bond formation by the reaction of potassium thiocyanate and aryl halides in the presence of a copper catalyst and a ligand in aqueous medium at 130 °C for 48 h. Tao et al. described the synthesis of diaryl sulfides catalyzed by CuI via cross-coupling between aryl halides and thioacetamide using Cs_2_CO_3_ as the base and DMSO–H_2_O as the solvent at 120 °C [44].

These recently developed protocols involve expensive catalytic systems and ligands [43], long reaction times [43–44], metal contamination of the final product, and non recyclability of the catalyst. This leads to increased costs as well as limiting the scope of the reaction. From the synthetic point of view, it is desirable to find novel recyclable catalytic systems, especially under ligand-free conditions, for the synthesis of such highly useful organic compounds.

In the past few years, considerable efforts have been made in the area of heterogeneous catalysis for various organic transformations. In general, heterogeneous catalysts offer higher surface area and lower coordinating sites, which are responsible for their higher catalytic activity [45–47]. Furthermore, heterogeneous catalysis has the advantage of high atom efficiency, easy product purification, and reusability of the catalyst. However, up until now, the investigation of nanoparticles as catalysts has been limited and will be widely studied in future.

## Results and Discussion

**Scheme 1:**
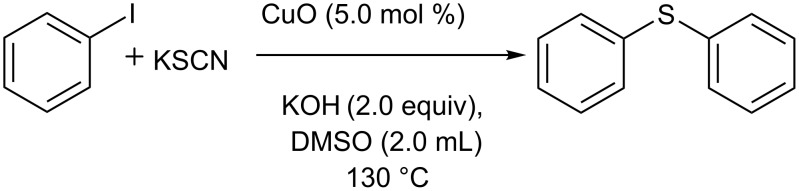
Synthesis of symmetrical aryl sulfides catalyzed by copper oxide nanoparticles.

In continuation of our investigations on metal-catalyzed cross-coupling reactions, we have explored the CuO-catalyzed synthesis of diaryl sulfides under ligand-free conditions (Scheme 1). To the best of our knowledge, this is the first recyclable copper oxide nanoparticle-catalyzed cross-coupling of aryl halides with potassium thiocyanate [46–51].

Initially, the reaction between potassium thiocyanate and iodobenzene was selected as the model reaction for optimizing the reaction conditions involving various copper sources, bases, solvents, and temperature (Table 1).

**Table 1:** Screening of copper sources for the cross-coupling reaction between iodo benzene and potassium thiocyanate.^a^

Entry	Catalyst	Solvent	Base	T (°)	Yield (%)^b^

1	CuO	DMSO	K_2_CO_3_	rt	0
2	CuO	DMSO	K_2_CO_3_	80	46
3	CuO	DMSO	K_2_CO_3_	130	51
4	CuO	DMSO	Cs_2_CO_3_	130	79
**5**	**CuO**	**DMSO**	**KOH**	**130**	**94**
6	CuO	DMSO	K_3_PO_4_	130	42
7	CuO	Toluene	KOH	130	trace
8	CuO	H_2_O	KOH	130	trace
9	CuO	PEG	KOH	130	60
10	CuO	DMF	KOH	130	70
11	–	DMSO	KOH	130	0^c^
12	CuO	DMSO	–	130	0^d^
13	CuCl_2_·2H_2_O	DMSO	KOH	130	83
14	CuSO_4_·5H_2_O	DMSO	KOH	130	71
15	Cu(OAc)·H_2_O	DMSO	KOH	130	79

^a^Reaction conditions: Iodobenzene (2.0 mmol), potassium thiocyanate (1.5 mmol), nano CuO (5.0 mol %), solvent (2.0 mL), base (2.0 equiv), 130 °C, 20 h. ^b^Isolated yield. ^c^In the absence of catalyst. ^d^In the absence of base.

First, several copper catalysts were screened (Table 1), and CuO was found to be promising for this tandem reaction (Table 1, entry 5). Amongst various bases screened, Cs_2_CO_3_ and KOH afforded the symmetrical aryl sulfides in excellent yields (Table 1, entries 4, 5). Other bases such as K_2_CO_3_ and K_3_PO_4_ gave lesser amounts of the desired product (Table 1, entries 3, 6). Among the solvents, DMSO yielded the best results (Table 1, entry 5) whereas PEG and DMF gave the products in moderate yields (Table 1, entries 9 and 10), whilst solvents such as toluene and water were ineffective (Table 1, entries 7 and 8). The coupling reaction did not occur in the absence of the catalyst (Table 1, entry 11) or base (Table 1, entry 12). When the reaction was conducted either at room temperature and 80 °C, no product was obtained or the yield was very low (Table 1, entries 1 and 2). The ideal temperature for the reaction was found to be 130 °C.

A study was conducted on C–S cross-coupling reaction using various sulfur sources under these conditions (Table 2). Among these sulfur surrogates potassium thiocyanate gave good yields in this C–S cross-coupling reaction.

**Table 2:** Nano CuO catalyzed C–S cross-coupling of iodobenzene with different sulfur sources.^a^

Entry	Aryl halide	Sulfur source	Yield (%)^b^

1	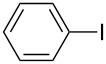	KSCN	94
2		NH_4_SCN	91
3		Na_2_S_2_O_3_	59

4	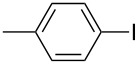	KSCN	91
5		NH_4_SCN	85
6		Na_2_S_2_O_3_	54

7	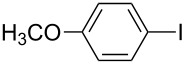	KSCN	92
8		NH_4_SCN	81
9		Na_2_S_2_O_3_	58

^a^Reaction conditions: Aryl halides (2.0 mmol), sulfur sources (1.5 mmol), nano CuO (5.0 mol %), DMSO (2.0 mL), KOH (2.0 equiv), 130 °C, 20 h, ^b^Isolated yield.

While expanding the scope of this nano CuO catalyzed tandem cross-coupling, the reaction of potassium thiocyanate with various aryl halides was examined under the optimized conditions. In general, all the reactions were very clean, and the symmetrical aryl sulfides were obtained in moderate to good yields. This protocol efficiently coupled iodobenzenes with electron donating groups (e.g., Me, OMe and Et) with potassium thiocyanate to produce the corresponding products in excellent yields (Table 3, entries 4, 6 and 8), whereas in the presence of an electron withdrawing group (CF_3_, NO_2_) a slight decrease in the yield of the diaryl sulfide (Table 3, entries 12 and 13) was observed. Under these reaction conditions, various hetero aromatic iodides were reacted with potassium thiocyanate and gave the corresponding diaryl sulfides in appreciable yields (Table 3, entries 15, 16 and 17). In case of the reactions of aromatic and hetero aromatic bromides with potassium thiocyanate, longer reaction times were required in order to obtain reasonable yields of diaryl sulfides (Table 3, entry 2, 5, 7 and 18). Iodobenzene was found to be a more reactive substrate than bromo, and chloro benzenes (Table 3, entries 1, 2 and 3).

**Table 3:** Copper oxide nanoparticles catalyzed synthesis of diaryl sulfides^a^.

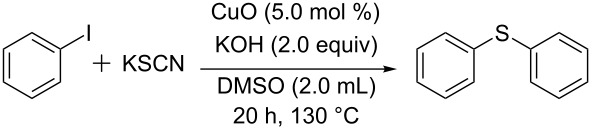			

Entry	Aryl halide	Product	Yield (%)^b^

1	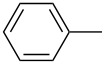	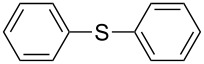	94
2	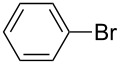	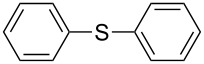	63^c^
3	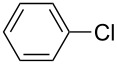	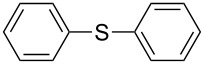	trace
4	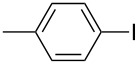	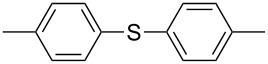	91
5	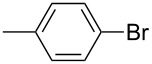	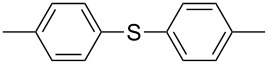	68^c^
6	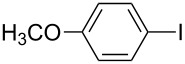	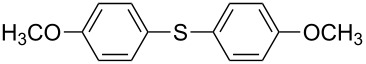	92
7	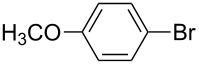	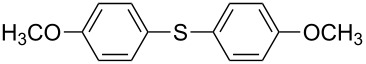	70^c^
8	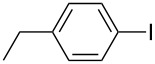	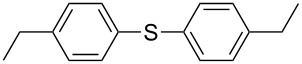	87
9	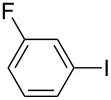	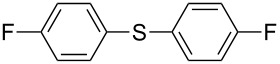	72
10	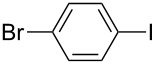	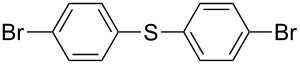	78
11	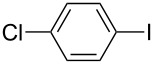	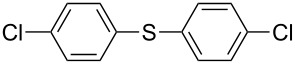	76
12	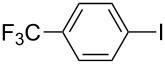	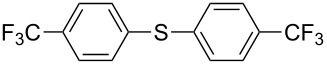	79
13	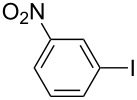	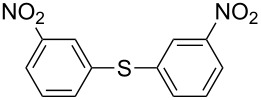	78
14	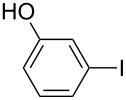	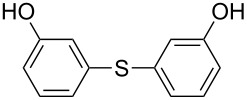	75
15	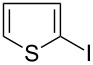	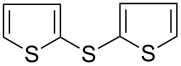	66
16	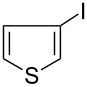	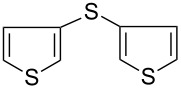	62
17	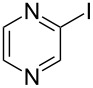	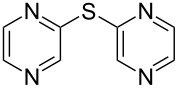	69
18	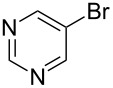	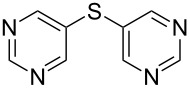	72^c^

^a^Reaction conditions: Aryl halides (2.0 mmol), potassium thiocyanate (1.5 mmol), nano CuO (5.0 mol %), DMSO (2.0 mL), KOH (2.0 equiv), 130 °C, 20 h. ^b^ Isolated yield. ^c^After 34 h.

## Conclusion

We have developed a CuO nanoparticles catalyzed synthesis of symmetrical diaryl sulfides via cascade reaction of aryl halides with potassium thiocyanate under ligand free conditions. The reaction avoids foul smelling thiols and the catalyst is economical, air stable, functions under ligand free conditions and is recyclable for up to four cycles without loss of catalytic activity [20,39–41] (Table 4).

**Table 4:** Copper oxide reusability of the catalyst.

Recylces	Yield (%)	Catalyst recovery (%)

Native	94	93
1	92	91
2	89	88
3	86	85

## Experimental

**General procedure for the synthesis of diaryl sulfides:** A mixture of aryl iodide (2.0 mmol), potassium thiocyanate (1.5 mmol), nano CuO (5.0 mol %), and KOH (2.0 equiv) was stirred at 130 °C under a N_2_ atmosphere in DMSO (2.0 mL). The progress of the reaction was monitored by TLC. When the reaction was complete, the reaction mixture was allowed to cool, a 1:1 mixture ethyl acetate and water (20 mL) was added and the CuO was removed by centrifugation. The organic layer was washed successively with brine and water, and dried with Na_2_SO_4_. The solvent and volatiles were completely removed under vacuum to give the crude product, which was purified by column chromatography on silica gel to yield the analytically pure product in up to 94% yield. The identity and purity of the product was confirmed by ^1^H and ^13^C NMR spectroscopy.

## Supporting Information

File 1 Experimental details and spectroscopic data for new compounds.
