# Supplementary material for: Nano copper oxide catalyzed synthesis of symmetrical diaryl sulfides under ligand free conditions
Source: Beilstein J Org Chem. 2011 Jun 30;7:886–91. doi: 10.3762/bjoc.7.101 (PMC3135086; doi:10.3762/bjoc.7.101)
Supplement: File 1 — Experimental details and spectroscopic data for new compounds. [file Beilstein_J_Org_Chem-07-886-s001.pdf]

Supporting Information

for

Nano copper oxide catalyzed synthesis of symmetrical diaryl sulfides under ligand free conditions

K. Harsha Vardhan Reddy, V. Prakash Reddy, A. Ashwan Kumar, G. Kranthi and Y.V.D. Nageswar*

Address: Organic Chemistry Divison-I, Indian Institute of Chemical Technology, Hyderabad-500 607, India

Email: Y.V.D. Nageswar* - dryvdnageswar@gmail.com

* Corresponding author

Experimental details and spectroscopic data for new compounds

Contents:

**General information**	S2
**General procedure for the synthesis of symmetrical diaryl sulfides**	S2
**Spectroscopic data**	S3
**References**	S7
**Copies of ^{1}H NMR and ^{13}C NMR of compounds**	S8

General information:

CuO nanoparticles (mean particle size 33 nm, surface area, 29 m^2/g and purity, 99.99%) were purchased from Sigma Aldrich. Analytical thin layer chromatography (TLC) was carried out using silica gel 60 F_{254} pre-coated glass plates. Visualization was accomplished with a UV lamp and I_2 stain. All products were characterized by NMR and MS. ^1H and ^{13}C NMR was recorded on 100, 200 and 300 MHz spectrometers, in $CDCl_3$ with TMS as the internal standard, chemical shifts are reported in parts per million (ppm, δ) downfield from tetramethylsilane.

General procedure for synthesis of symmetrical diaryl sulfides:

A mixture of the aryl iodide (2.0 mmol), potassium thiocyanate (1.5 mmol), nano CuO (5.0 mol %), and KOH (2.0 equiv) was stirred at 130 °C under a N_2 atmosphere in DMSO (2.0 mL). The progress of the reaction was monitored by TLC. When the reaction was complete, the reaction mixture was allowed to cool, a 1:1 mixture ethyl acetate and water (20 mL) was added, and CuO was removed by centrifugation. The organic layer was washed successivelywith brine and water, and dried with Na_2SO_4. The solvent and volatiles were completely removed under vacuum to give the crude product, which was purified by column chromatography on silica gel to give the analytically pure diaryl sulfide (94%).

Recycling of the catalyst:

After the reaction was complete, the reaction mixture was allowed to cool, a 1:1 mixture of ethyl acetate/water (2.0 mL) was added, and CuO was removed by centrifugation. After each cycle, the catalyst was recovered by simple centrifugation, washed with deionized water and ethyl acetate, and then dried in vacuo. The recovered nano-CuO was used directly in the next cycle.

Spectroscopic data:

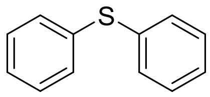

Diphenylsulfane (Table 3, entry1) [1]:

Colorless oil

^{1}**H NMR (**300 MHz, $CDCl_3$, TMS): δ =7.32–7.16 (m, 10H).

^{13}**C NMR** (75 MHz, $CDCl_3$, TMS): δ = 135.7, 131.0, 129.1, 127.0.

Mass (EI): *m/z* 186 $[M]^+$

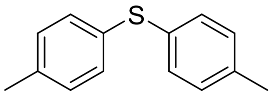

Di-*p*-tolylsulfane (Table 3, entry 4) [1]:

Yellow oil

^{1}**H NMR** (200 MHz, $CDCl_3$, TMS): δ = 7.21 (d, 4H, *J* = 8.01 Hz), 7.06 (d, 4H, *J* = 8.00 Hz), 2.32 (s, 6H).

^{13}**C NMR** (50 MHz, $CDCl_3$, TMS): δ = 136.7, 132.81, 131.0, 129.8, 96.1.

Mass (EI): *m/z* 214 $[M]^+$

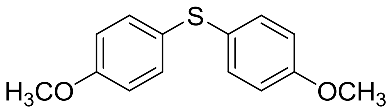

Bis(4-methoxyphenyl)sulfane (Table 3, entry 6) [1]:

Colorless oil

^{1}**H NMR** (300 MHz, $CDCl_3$, TMS): δ = 7.50–7.22 (m, 4H), 6.82 (d, 4H, *J* = 8.60 Hz), 3.74 (s, 6H).

^{13}**C NMR** (75 MHz, $CDCl_3$, TMS): δ = 158.9, 132.6, 114.6, 114.5, 55.2.

Mass (EI): *m/z* 246 $[M]^+$

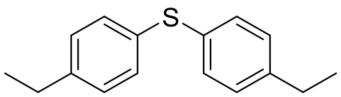

Bis(4-ethylphenyl)sulfane (Table 3, entry 8):

Colorless oil

IR (neat): ν = 2962.71, 2924.56, 2858.78, 1488.79, 1455.90, 1403.36, 822.82cm^{-1}

^{1}**H NMR** (300 MHz, $CDCl_3$, TMS): δ = 7.21(d, 4H, *J* = 7.80 Hz), 7.07(d, 4H, *J* = 7.81 Hz), 2.62–2.52 (m, 4H), 1.26 (t, 6H, *J* = 7.80 Hz).

^{13}**C NMR** (75 MHz, $CDCl_3$, TMS): δ = 143.1, 132.7, 131.0, 128.6, 28.3, 15.4.

Mass (EI): *m*/*z* 242 $[M]^+$

Anal. calcd for: ($C_{18}H_{18}S$) C, 79.29; H, 7.49; S, 13.23.

Found: C, 79.22; H, 7.42; S, 13.19.

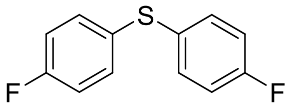

Bis(4-fluorophenyl)sulfane (Table 3, entry 9):

Colorless oil

^{1}**H NMR** (300 MHz, $CDCl_3$, TMS): δ = 7.31–7.24 (m, 2H), 7.12–7.09 (m, 2H), 7.03–6.89 (m, 4H).

^{13}**C NMR** (300 MHz, $CDCl_3$, TMS): δ = 165.4, 160.4, 137.0, 130.6, 126.7, 118.1, 117.6, 114.7, 114.3.

Mass (EI): *m*/*z* 222 $[M]^+$

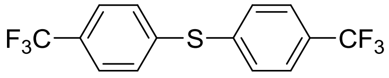

Bis(4-(trifluoromethyl)phenyl)sulfane (Table 3, entry 10):

Colorless oil

IR (neat): ν = 2925.01, 1586.43, 1471.85, 1217.41, 879.81, 777.01, 679.82cm^{-1}

^{1}H NMR (300 MHz, $CDCl_3$, TMS): δ = 7.80–7.34(m, 8H).

^{13}C NMR (75 MHz, $CDCl_3$, TMS): δ = 140.9, 136.4, 134.2, 129.8, 127.8, 124.4, 121.8.

Mass (EI): *m*/*z* 322 $[M]^+$

Anal. calcd for: ($C_{14}H_8F_6S$) C, 52.18; H, 2.50; S, 9.95.

Found: C, 52.16; H, 2.47; S, 9.93.

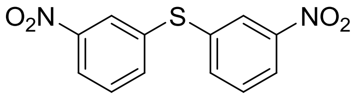

Bis(3-nitrophenyl)sulfane (Table 3, entry 11):

Pale yellow oil

IR (neat): ν = 2922.71, 2857.70, 1523.17, 1344.41, 875.13, 730.92cm^{-1}

^{1}H NMR (300 MHz, $CDCl_3$, TMS): δ = 8.19–8.15 (m, 4H), 7.65 (d, 2H, *J* = 8.31 Hz), 7.55 (t, 2H, *J* = 8.30 Hz).

^{13}C NMR (75 MHz, $CDCl_3$, TMS): δ =148.8, 136.7, 130.7, 125.6, 122.7.

Mass (EI): *m*/*z* 276 $[M]^+$

Anal. calcd for: ($C_{12}H_8N_2O_4S$) C, 52.17; H, 2.92; S, 11.61; N, 10.14.

Found: C, 52.12; H, 2.90; S, 11.58; N, 10.11.

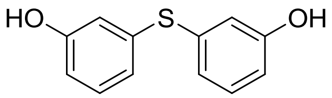

Bis(3-hydroxyphenyl)sulfane (Table 3, entry 12):

Colorless oil

^{1}H NMR (300 MHz, $CDCl_3$, TMS): δ = 7.32–7.13 (m, 2H), 6.97–6.70 (m, 6H), 5.51 (bs, 2H)

^{13}C NMR (75 MHz, $CDCl_3$, TMS): δ = 156.0, 136.7, 130.2, 128.3, 123.4, 117.6, 114.3.

Mass (EI): *m*/*z* 218 $[M]^+$

Anal. calcd for: ($C_{12}H_{10}O_2S$) C, 66.03; H, 4.62; S, 14.69.

Found: C, 66.01; H, 4.60; S, 14.64.

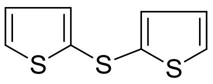

Dithiophen-2-ylsulfane (Table 3, entry 13):

Colorless oil

^{1}H NMR (300 MHz, $CDCl_3$, TMS): δ = 7.63–7.53 (m, 1H), 7.45–7.34 (m, 2H), 7.27–7.11 (m, 3H).

Mass (EI): *m/z* 197 $[M]^+$

Anal. calcd for: ($C_8H_6S_3$) C, 48.45; H, 3.05; S, 48.50.

Found: C, 48.42; H, 2.99; S, 48.47.

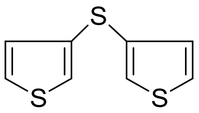

Dithiophen-3-ylsulfane (Table 3, entry14):

Yellow oil

IR (neat): ν = 2921.94, 1638.07, 1467.60, 1398.86, 839.79, 699.98cm^{-1}

^{1}H NMR (300 MHz, $CDCl_3$, TMS): δ = 7.31–7.25 (m, 2H), 7.17–7.11 (m, 2H), 6.96–6.94 (m, 2H).

^{13}C NMR (75 MHz, $CDCl_3$, TMS): δ = 129.6, 126.4, 124.7.

Mass (EI): *m/z* 197 $[M]^+$

Anal. calcd for: (C_8H_6S) C, 48.45; H, 3.05; S, 48.50.

Found: C, 48.42; H, 3.02; S, 48.47.

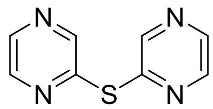

Dipyrazin-2-ylsulfane (Table 3, entry 15):

Colorless solid

M. P. : 90 °C

IR (neat): ν = 2923.46, 1454.73, 1387.00, 1119.88, 1007.66, 834.25cm^{-1}

^{1}**H NMR** (300 MHz, $CDCl_3$, TMS): δ =8.70–8.66 (m, 2H), 8.46–8.40 (m, 4H).

^{13}**C NMR** (75 MHz, $CDCl_3$, TMS): δ = 146.68, 144.67, 142.61.

Mass (EI): *m*/*z* 190 $[M]^+$

Anal. calcd for: ($C_8H_6N_4S$) C, 50.51; H, 3.18; S, 16.86.

Found: C, 50.50; H, 3.16; S, 16.85.

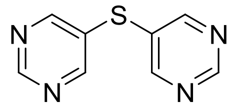

Dipyrimidin-5-ylsulfane (Table 3, entry 16):

Colorless oil

^{1}**H NMR** (300 MHz, $CDCl_3$, TMS): δ = 9.15 (s, 2H), 8.74 (s, 4H).

^{13}**C NMR** (75 MHz, $CDCl_3$, TMS): δ = 158.6, 157.7, 129.8.

Mass (EI): *m*/*z* 190$[M]^+$

Anal. calcd for: ($C_8H_6N_4S$) C, 50.51; H, 3.18; S, 16.86.

Found: C, 50.49; H, 3.17; S, 16.85.

References

(1) Ke, F.; Qu, Y.; Jiang, Z.; Li, Z.; Wu, D.; Zhou, X.; *Org. Lett.*, 2011, *13*, 454–457. DOI: 10.1021/ol102784c.

Copies of ^{1}H NMR and ^{13}C NMR of compounds:

Diphenylsulfane (Table 3, entry 1):

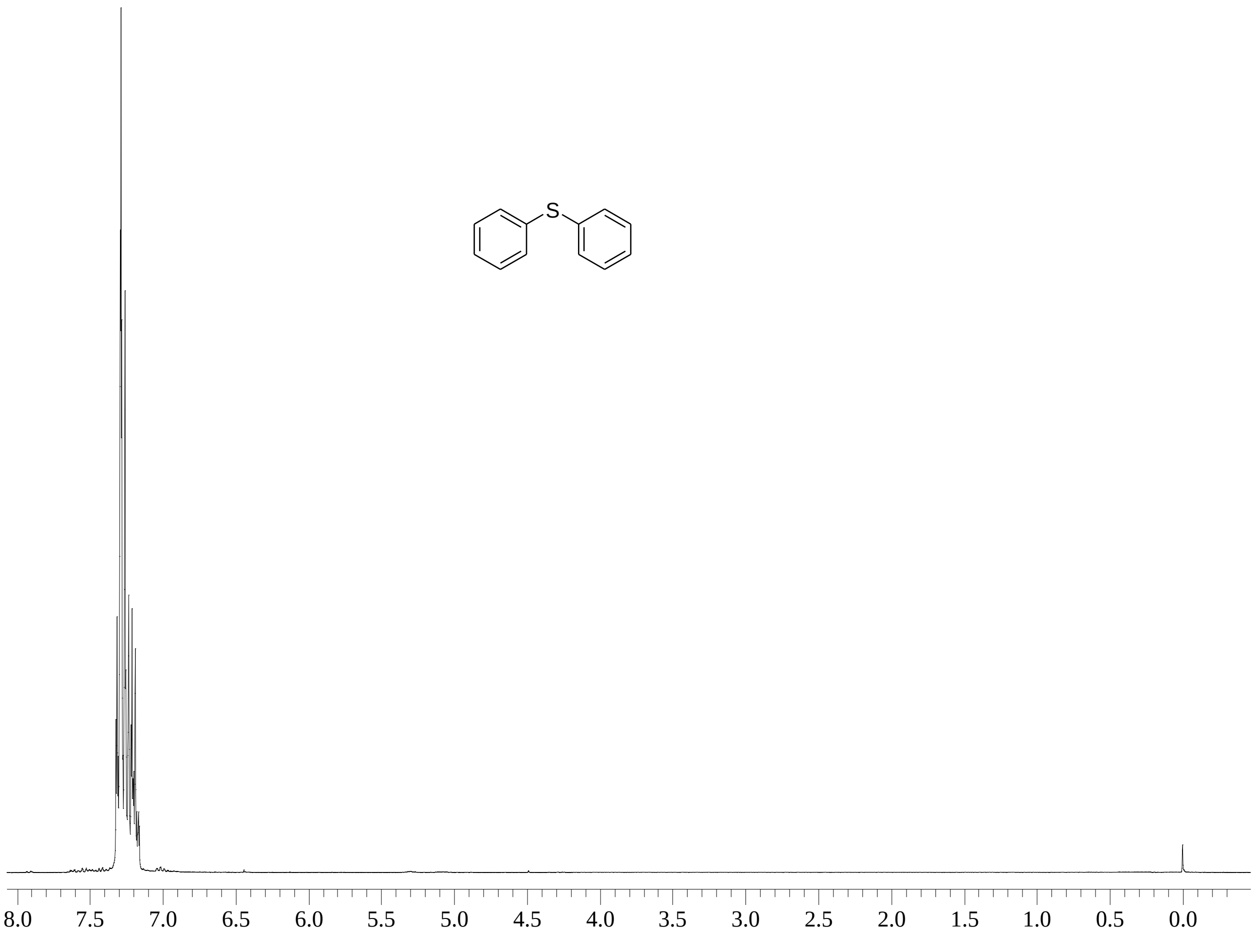

Di-*p*-tolylsulfane (Table 3, entry 3):

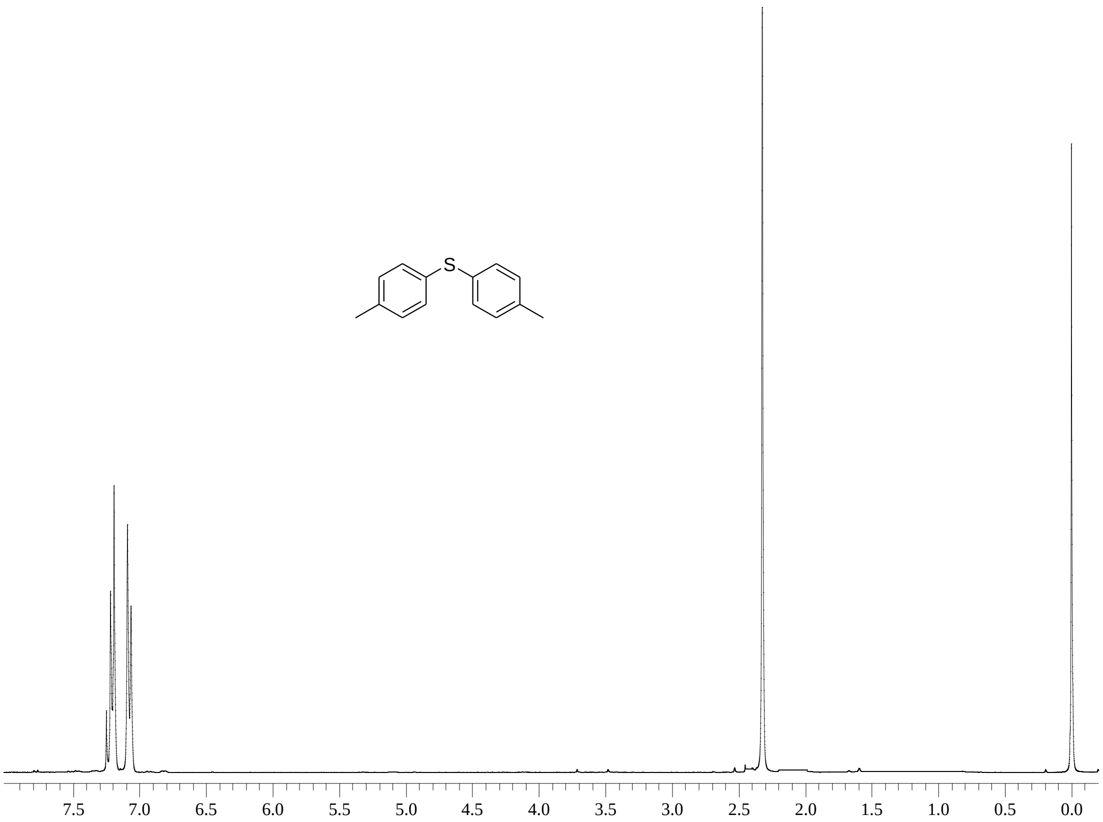

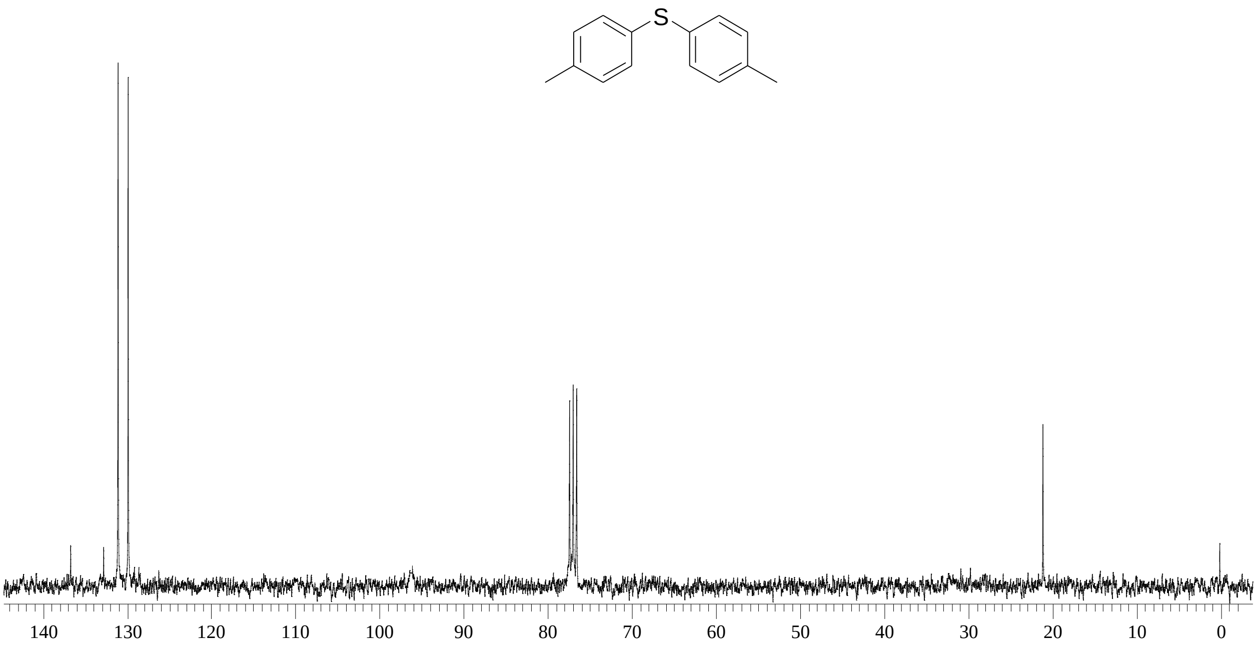

Bis(4-methoxyphenyl)sulfane (Table 3, entry 6):

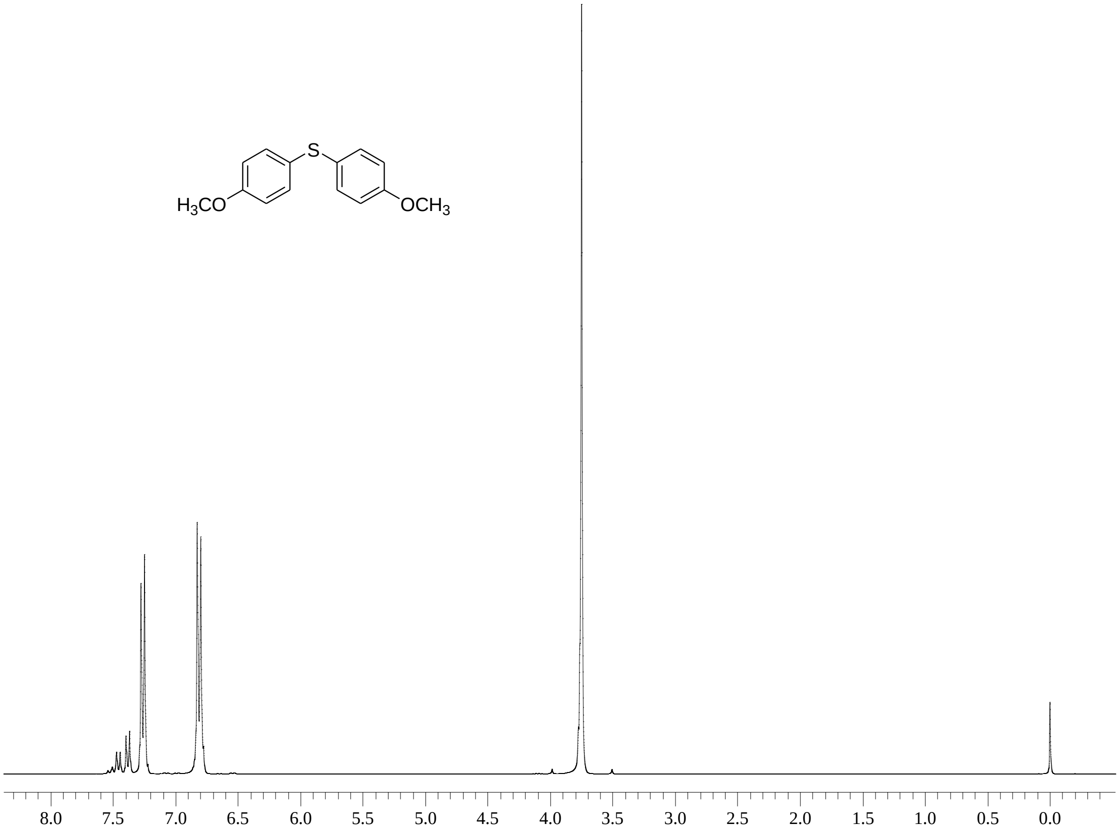

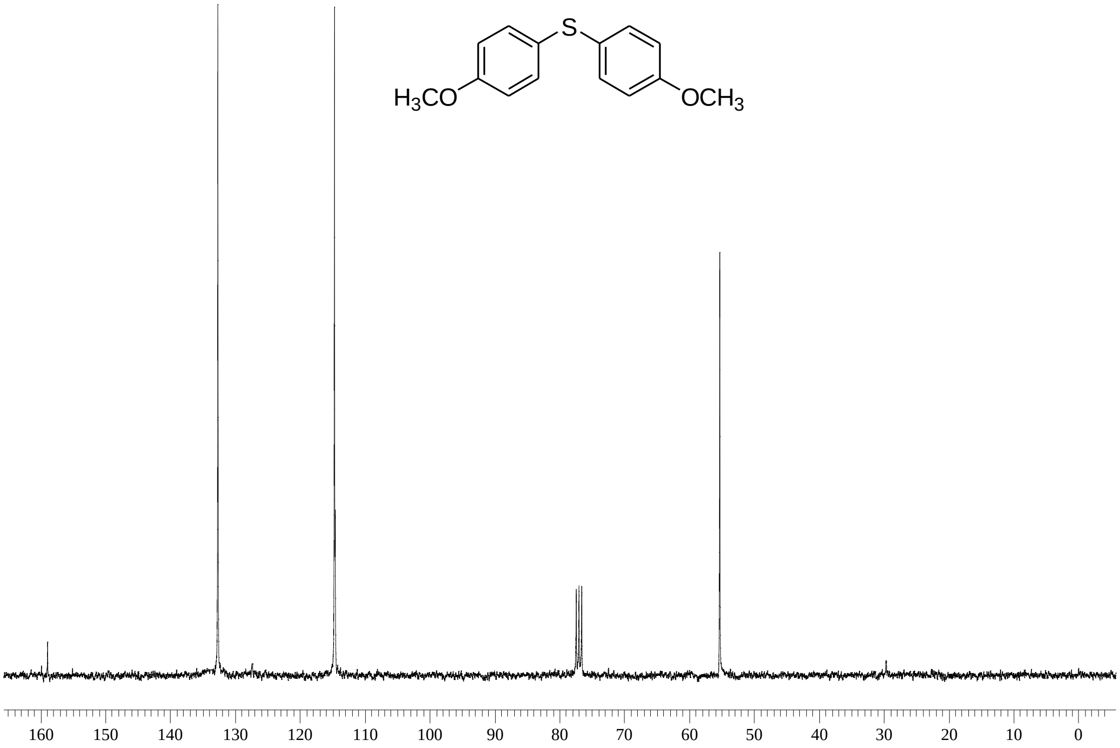

Bis(4-ethylphenyl)sulfane (Table 3, entry 8):

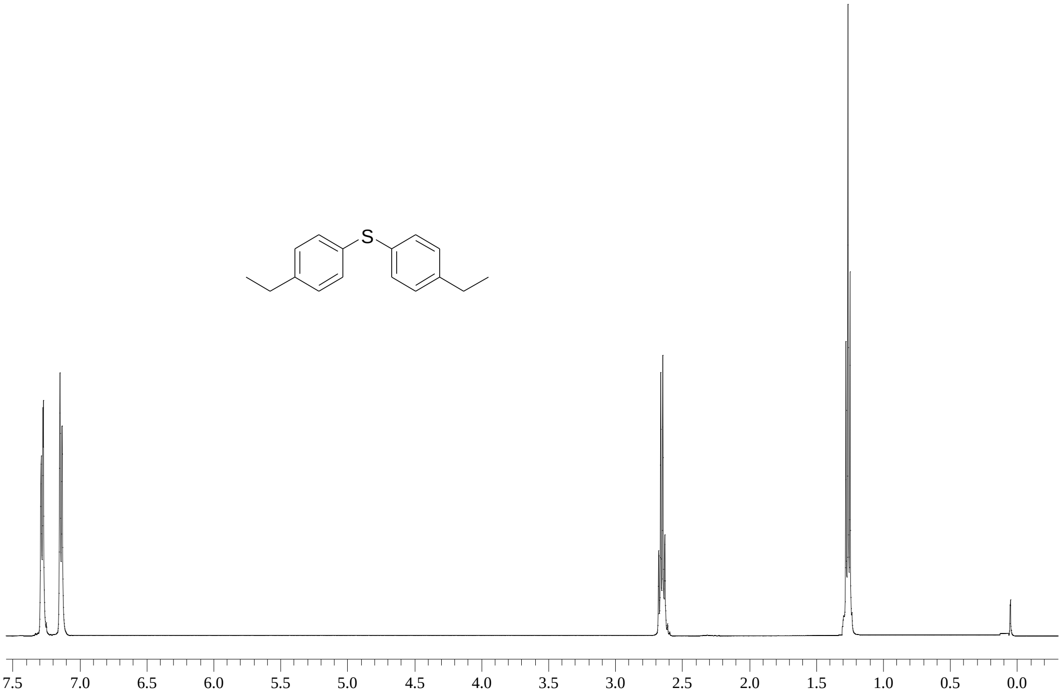

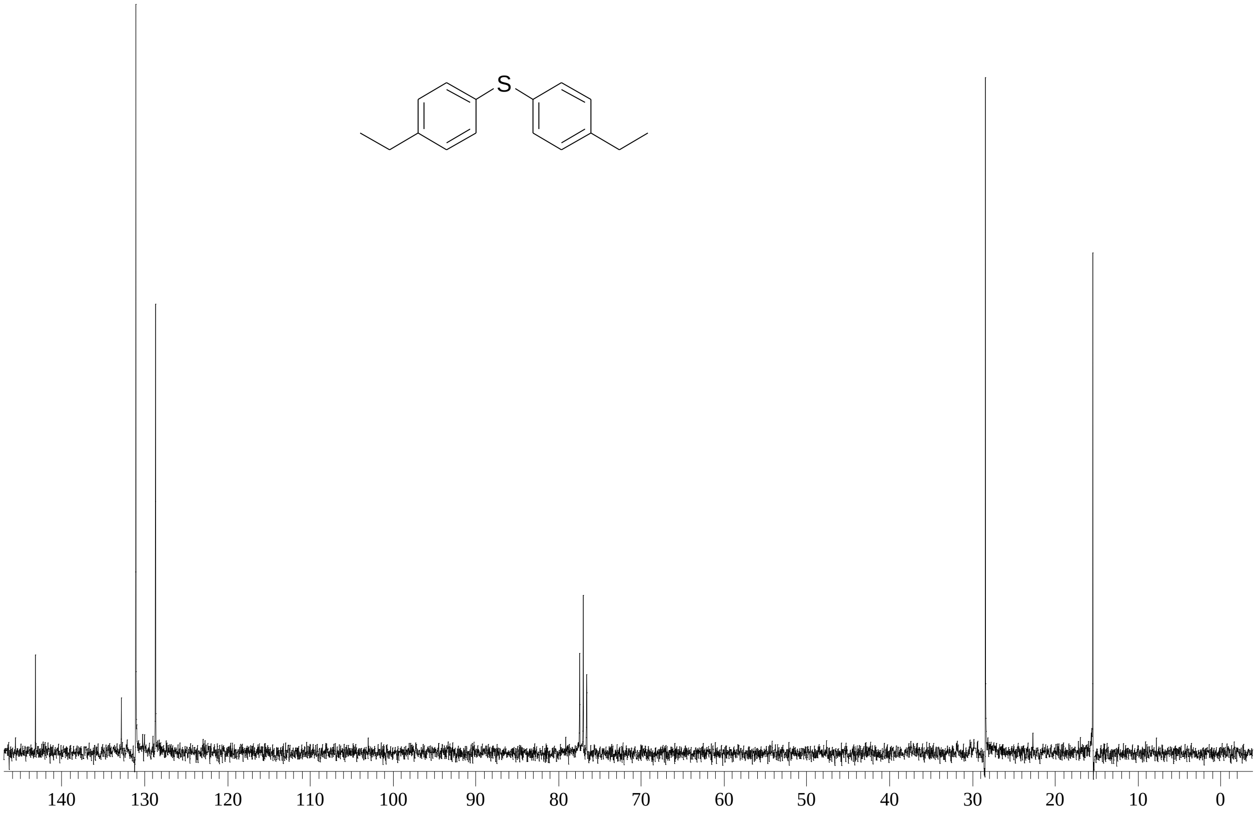

Bis(4-fluorophenyl)sulfane (Table 3, entry 9):

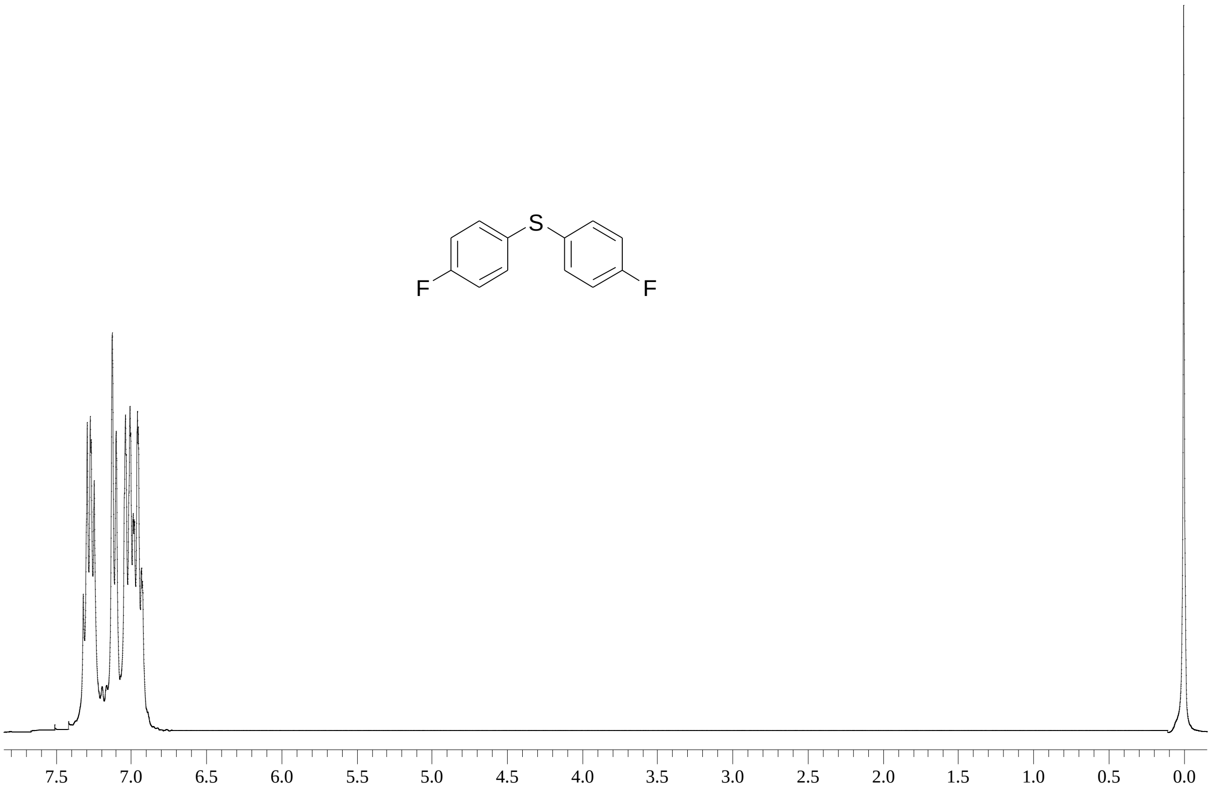

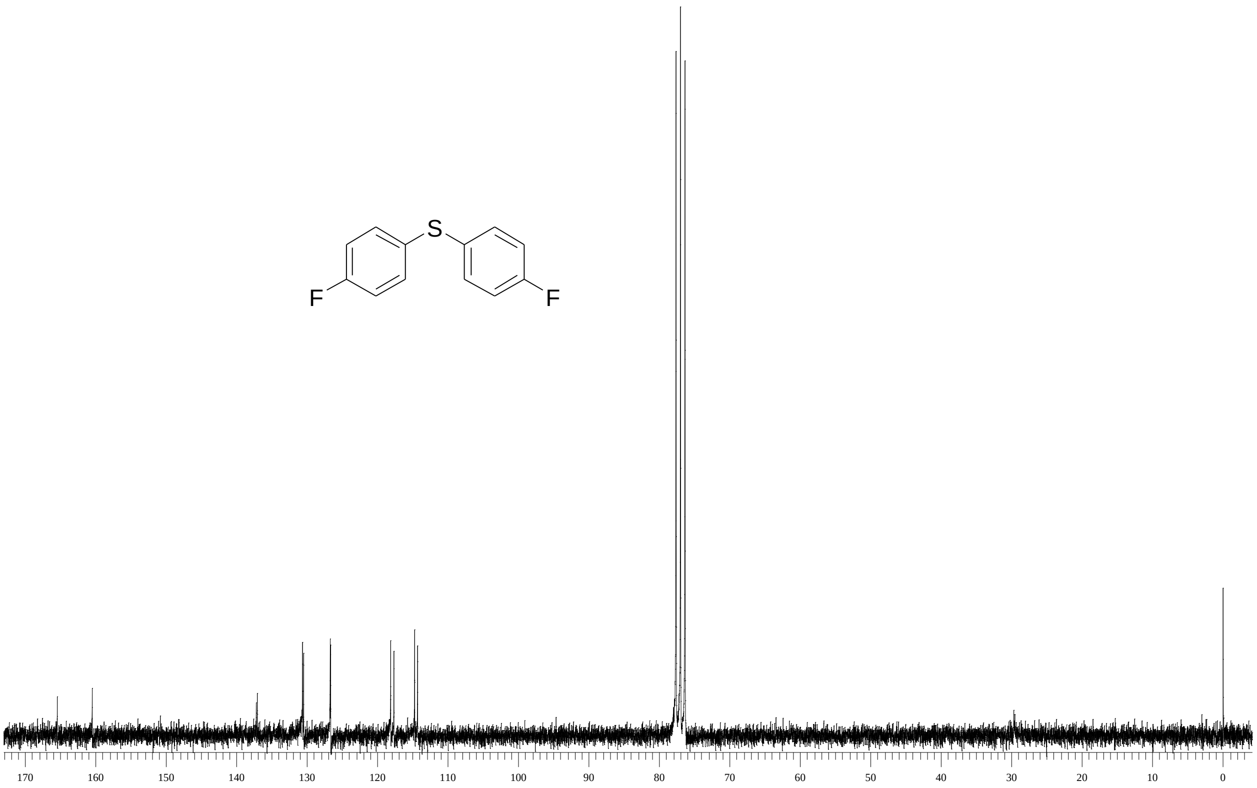

Bis(4-(trifluoromethyl)phenyl)sulfane (Table 3, entry 10):

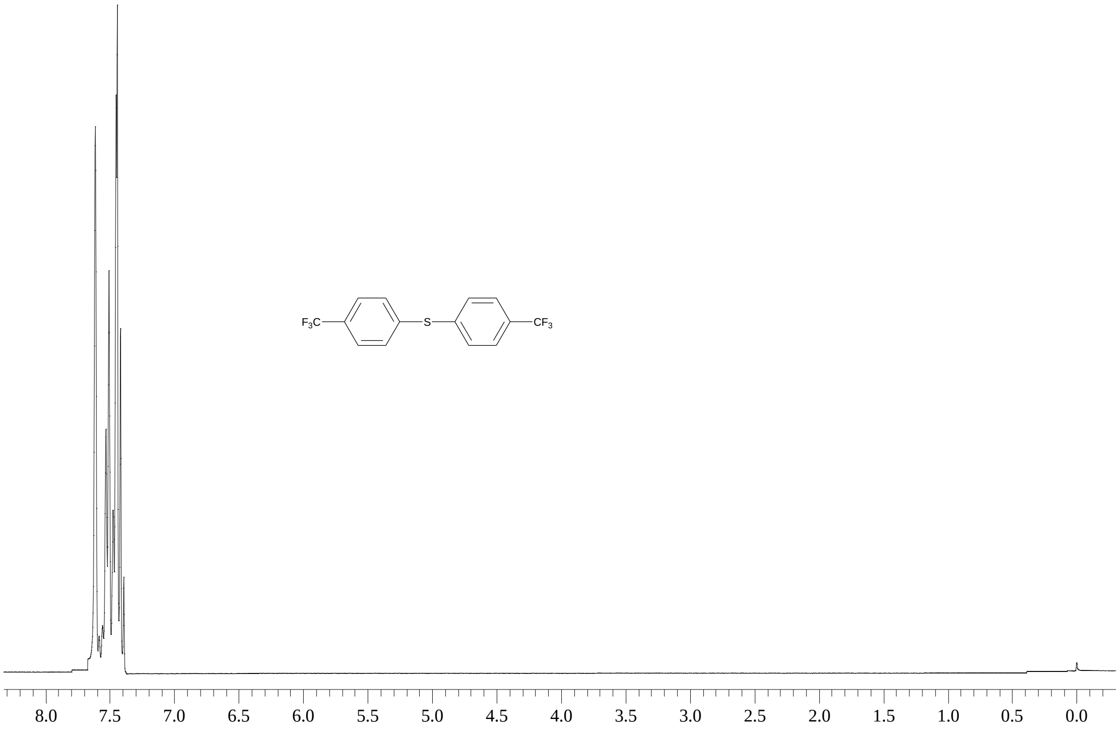

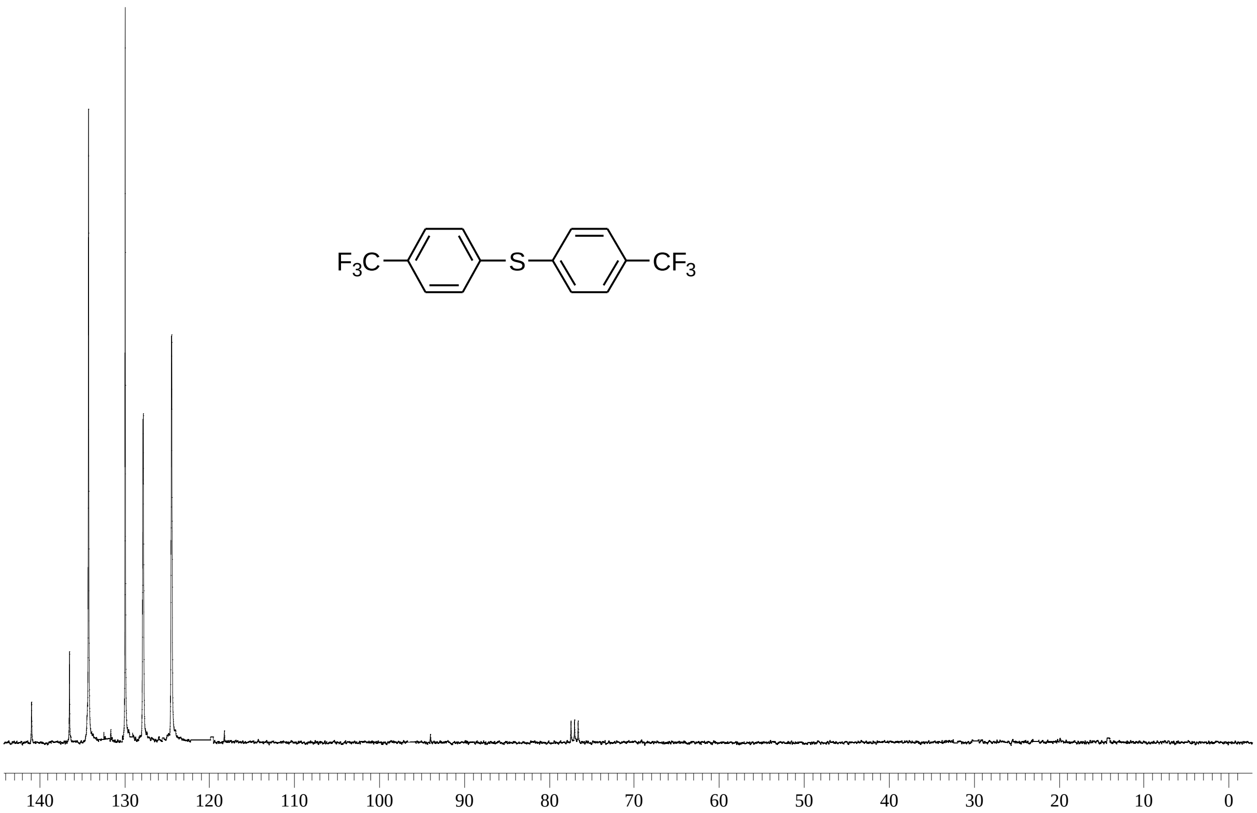

Bis(3-nitrophenyl)sulfane (Table 3, entry 11):

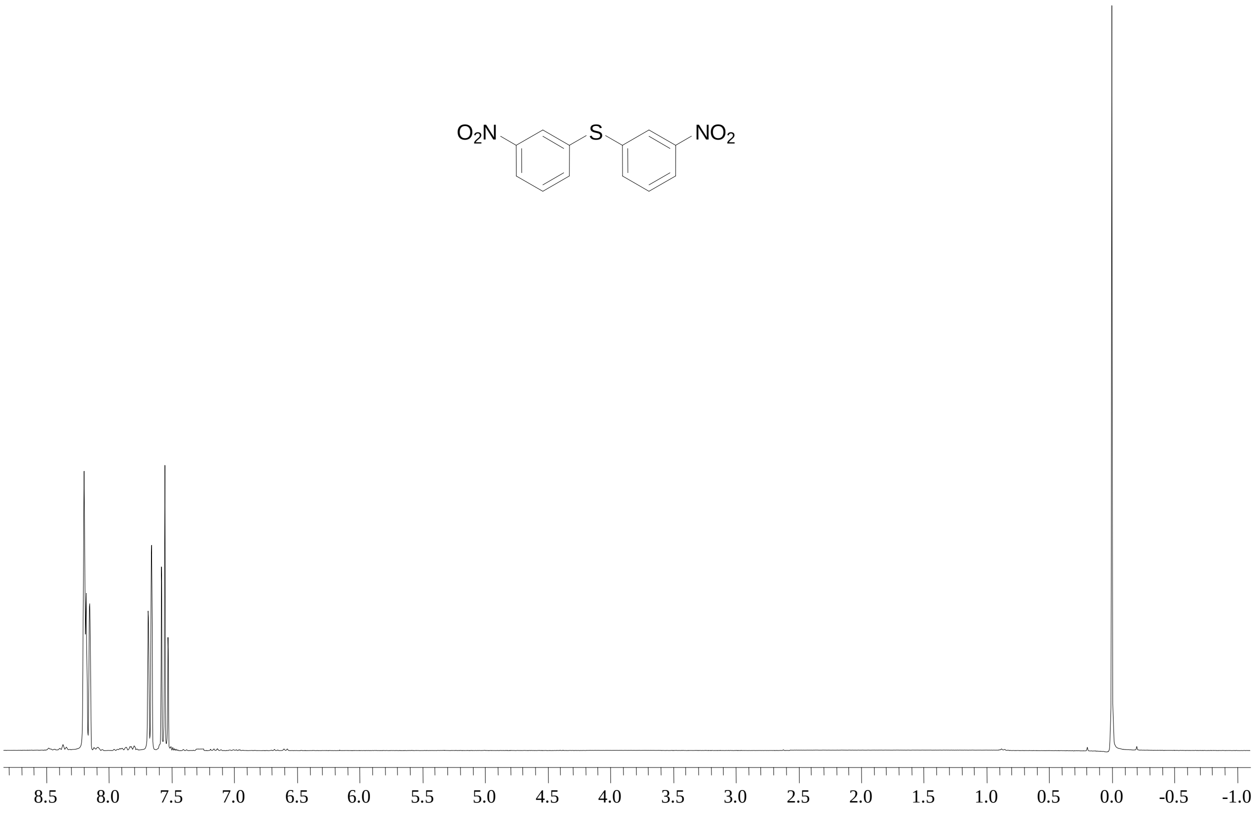

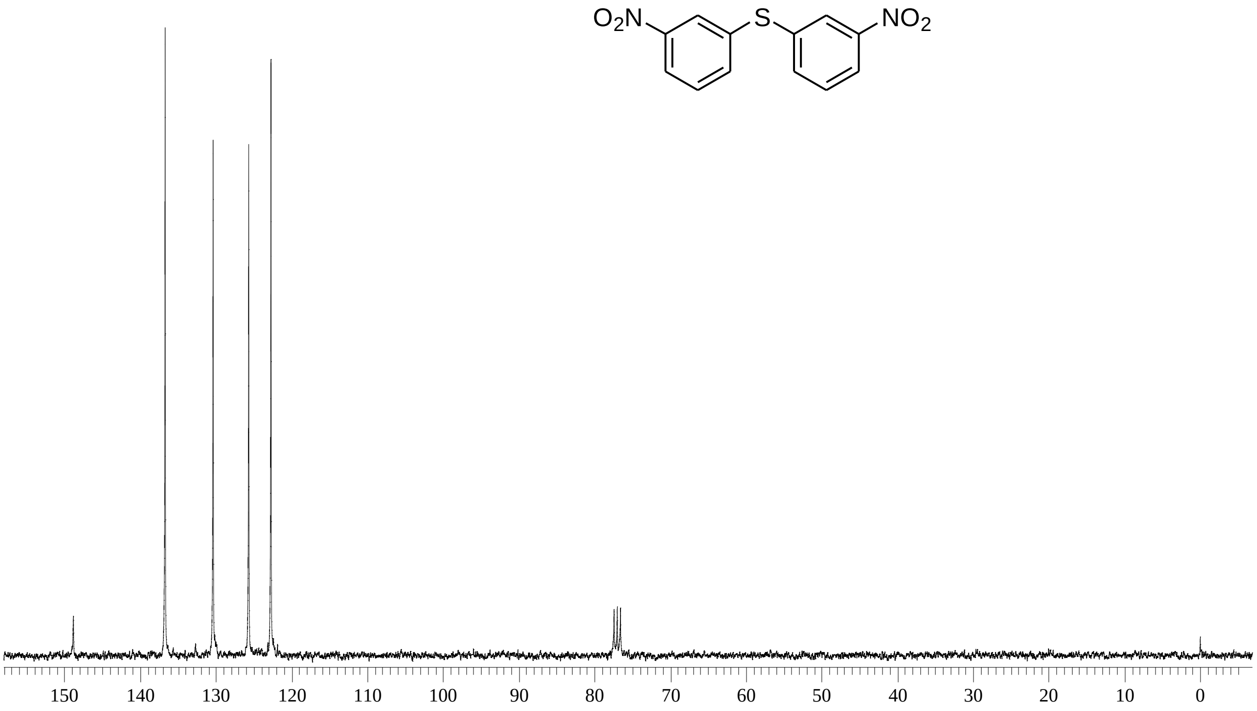

Bis(3-hydroxyphenyl)sulfane (Table 3, entry 12):

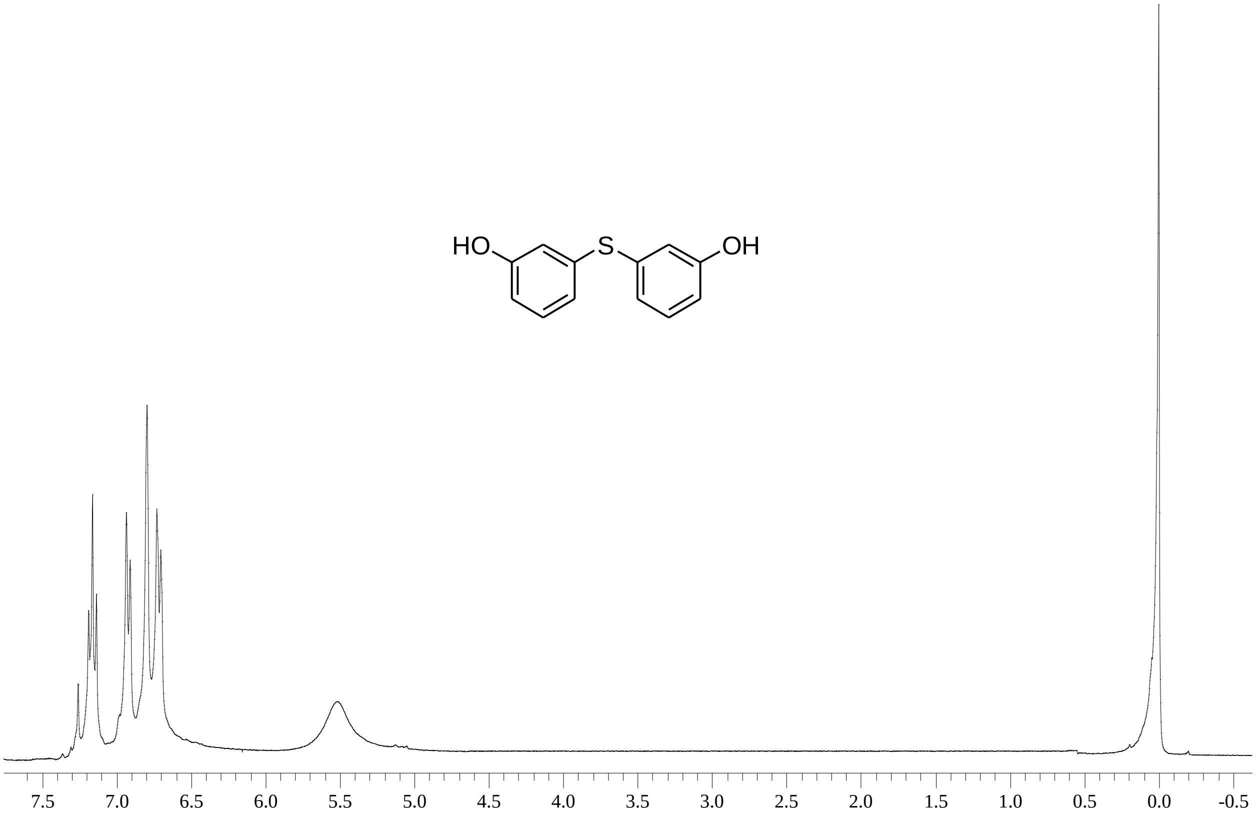

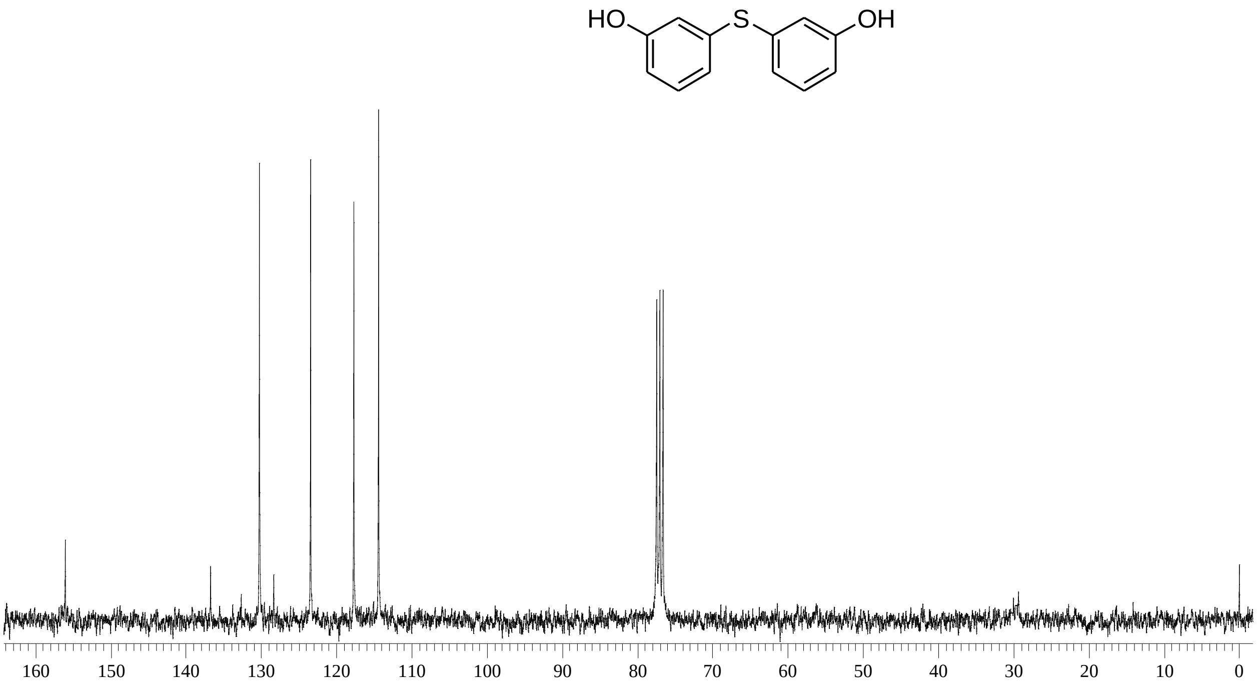

Dithiophen-2-ylsulfane (Table 3, entry 13):

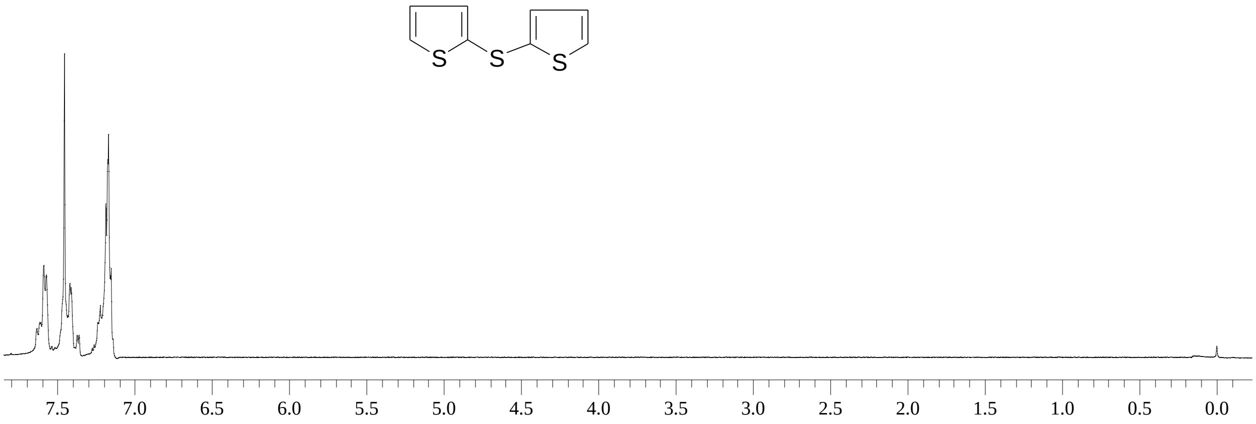

Dithiophen-3-ylsulfane (Table 3, entry 14)

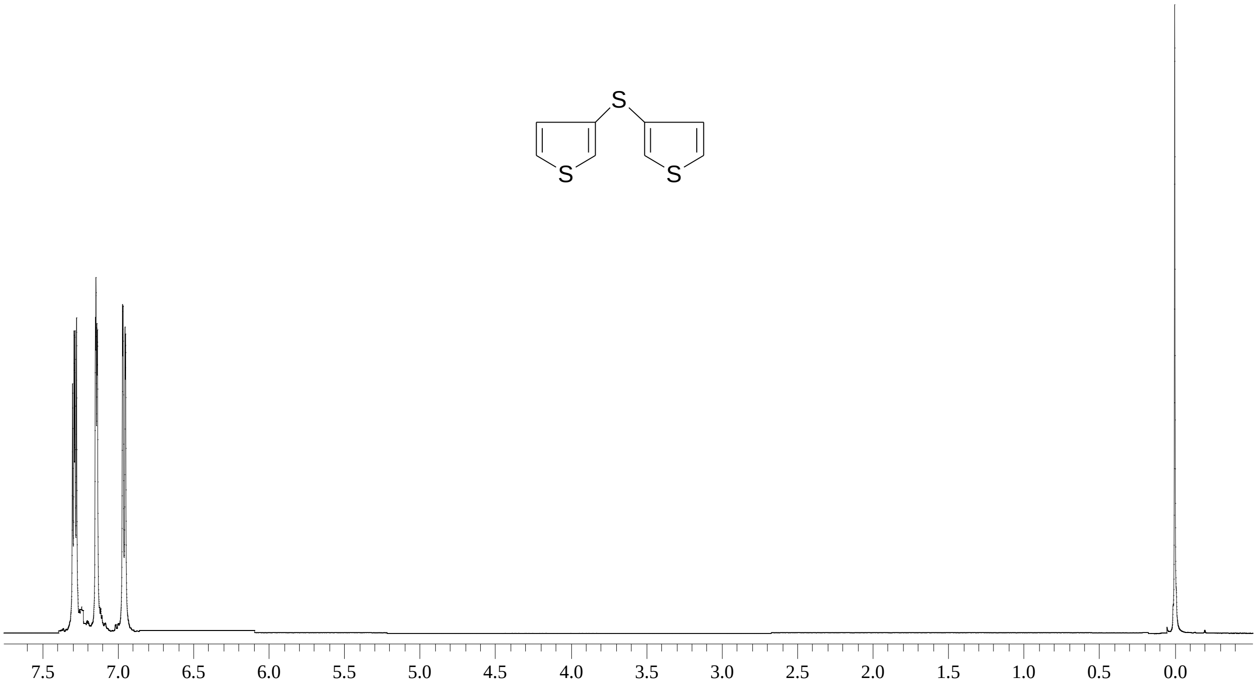

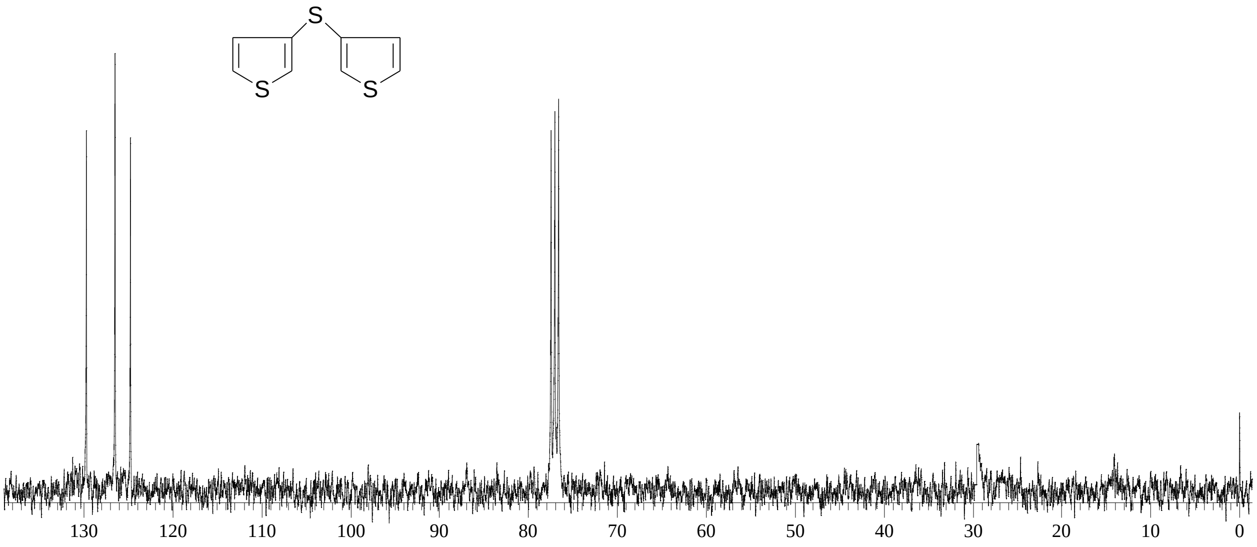

Dipyrazin-2-ylsulfane (Table 3, entry 15):

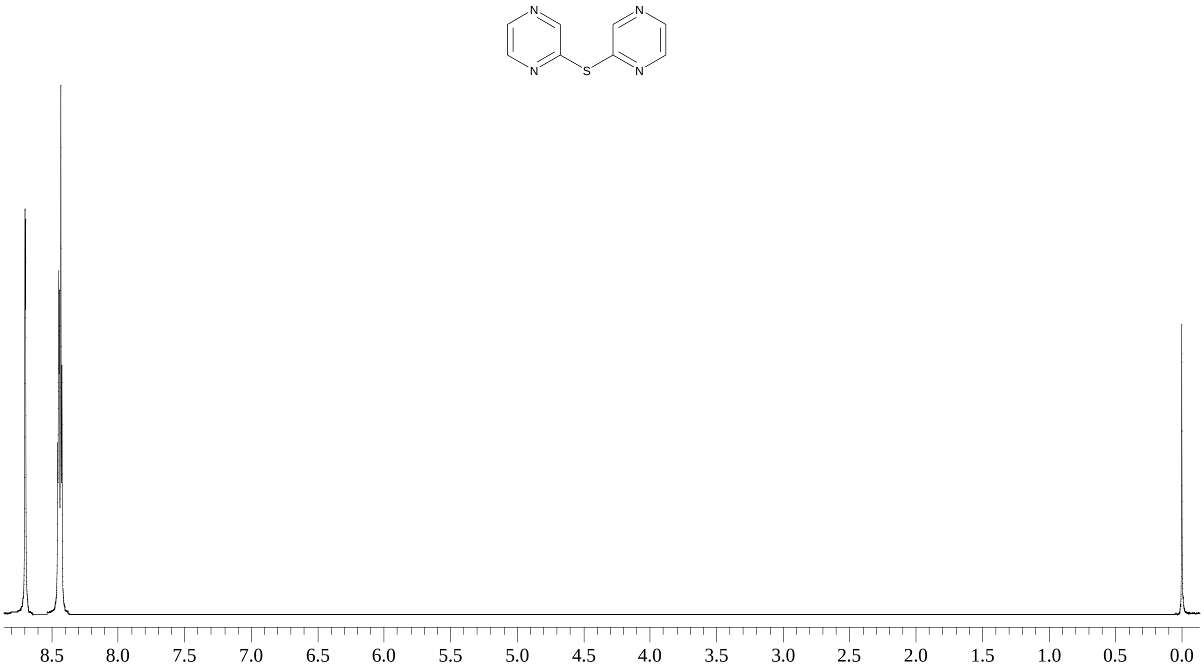

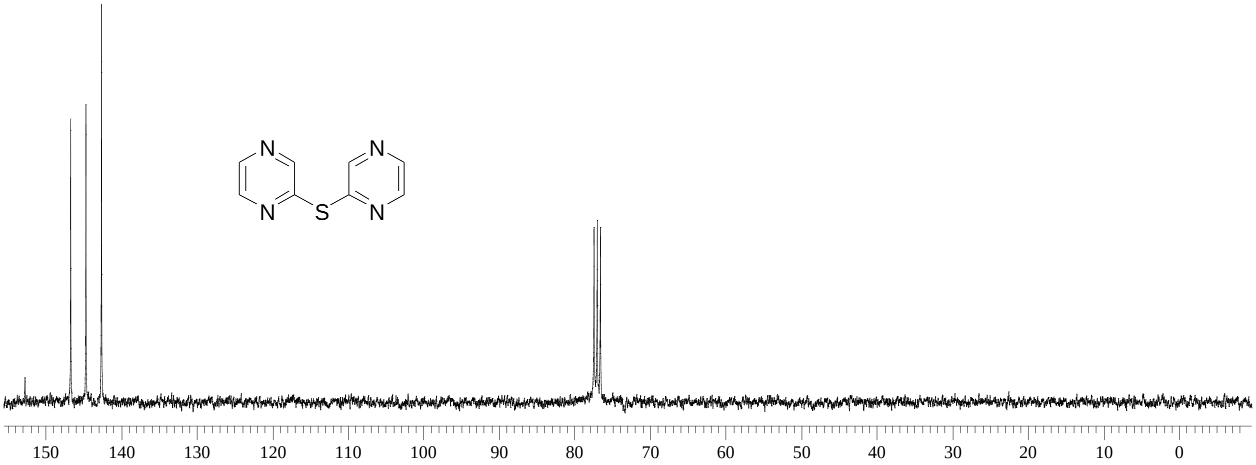

Dipyrimidin-5-ylsulfane (Table 3, entry 16):

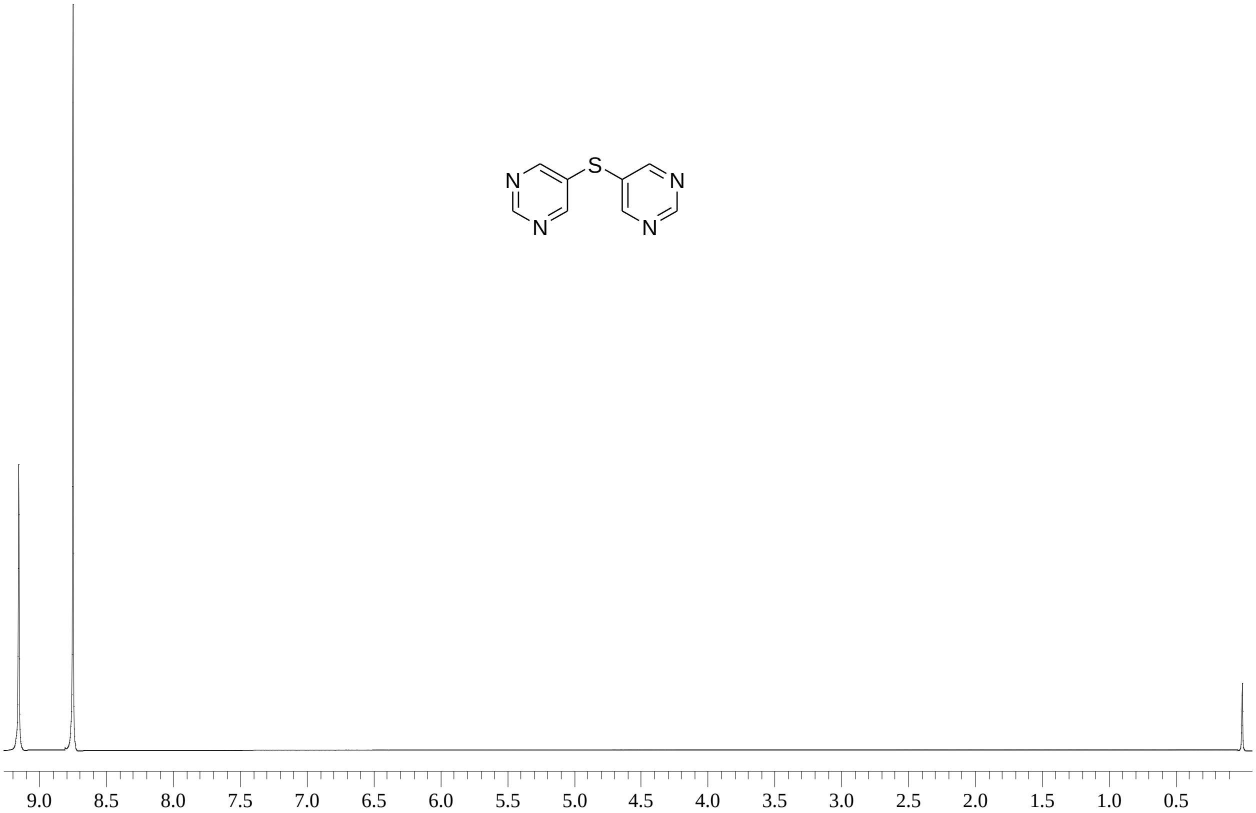

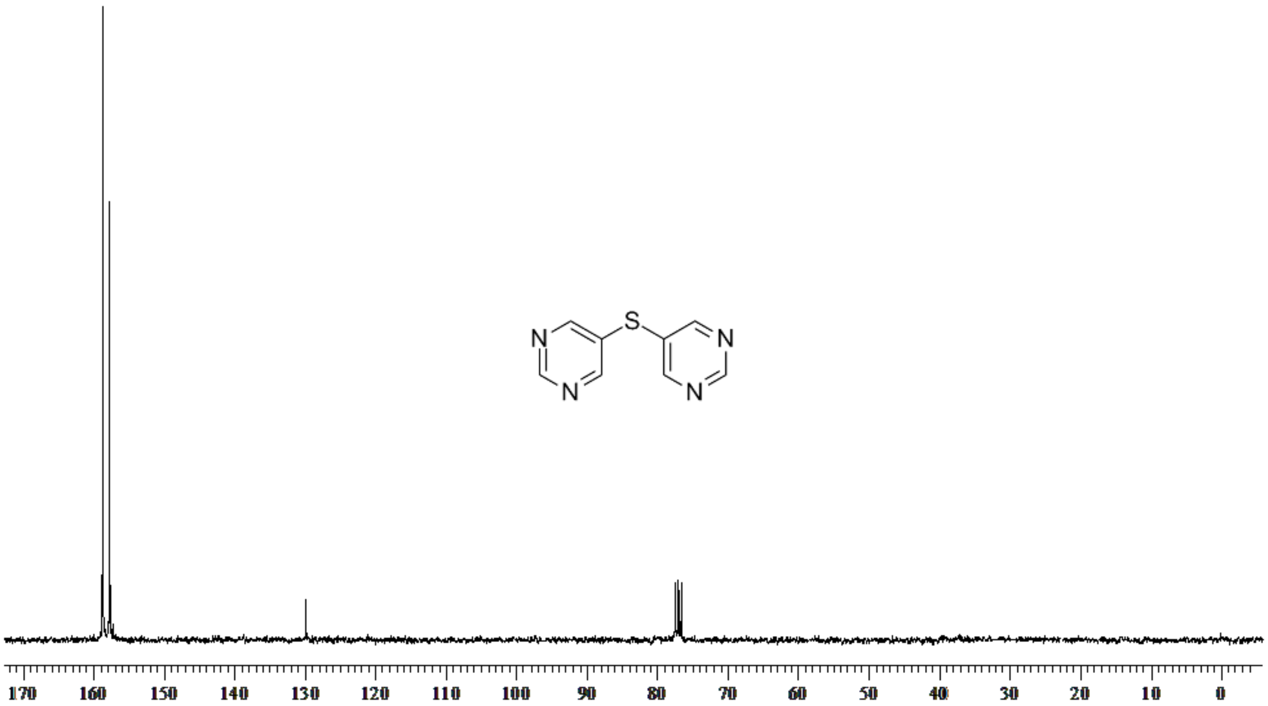

170 160 150 140 130 120 110 100 90 80 70 60 50 40 30 20 10 0
